# Perceived Barriers of Using Modern Family Planning Methods among Women in Jordan: A Qualitative Study

**DOI:** 10.30476/ijcbnm.2021.88675.1531

**Published:** 2021-10

**Authors:** Khulood K. Shattnawi, Yousef S. Khader, Nihaya Al-Sheyab, Mohammad Alyahya, Kelley Ready, Yara A. Halasa-Rappel, Heath Prince

**Affiliations:** 1 Department of Child Health Nursing, Faculty of Nursing, Jordan University of Science and Technology, Jordan; 2 Department of Public Health, Faculty of Medicine, Jordan University of Science and Technology, Jordan; 3 Department of Allied Medical Sciences, Faculty of Applied Medical Sciences, Jordan University of Science and Technology, Jordan; 4 Department of Health Management and Policy, Faculty of Medicine, Jordan University of Science and Technology, Jordan; 5 Ray Marshall Center for the Study of Human Resources, Lyndon B. Johnson School of Public Affairs, The University of Texas at Austin, USA; 6 Commonwealth Medicine, University of Massachusetts Medical School, Senior Project Director, University of Massachusetts, USA

**Keywords:** Barriers, Family planning methods, Jordan, Qualitative study, Women

## Abstract

**Background:**

Some cultural and social factors may discourage the use of modern family planning (MFP) methods. The purpose of this study was to better understand the barriers and social norms that might affect women’s ability to take optimal advantage of the free family planning services offered by the Jordanian Ministry of Health (MOH).

**Methods:**

Using a qualitative descriptive design, 7 focus group discussions were conducted from January to February 2018, with a purposive sample of 52 married women. Each group consisted of 6-12 participants. Ethical approvals were obtained. Data were analysed using inductive thematic analysis.

**Results:**

Data analysis revealed three main themes and four subthemes. The first theme ‘conforming to social and cultural norms’ included the following subthemes: ‘to conform to family and social pressure to bear children’ and ‘to prioritize having male children’. The second theme ‘unmet needs in expected family planning counselling’ included the following subthemes: ‘need for consistency across providers in family planning counselling’, and ‘need for follow-up counselling’. The third theme was the ‘undesirable side-effects’ of the MFP methods, which included both the ‘experienced’ and the ‘anticipated’ side effects.

**Conclusion:**

This study identified a number of women’s perceived barriers to using MFP methods. These included conforming to the social pressure, inconsistency of the counselling process, and undesirable side effects. Their perspectives should be carefully addressed in any family planning program.

## INTRODUCTION

The use of modern family planning (MFP) methods is widespread in Jordan and they are offered free of charge regardless of the person’s insurance type. The percentage of childbearing age women who use MFP methods has increased only from 41% in 2002 to 42% in 2012, and then declined to 37% in 2017-2018. ^1^ According to the Jordan Family and Health Survey 2017-2018, of currently married women in Jordan, 51.8% are using a type of contraceptive method; modern birth control methods are used by 37.4%, while 14.4% use “traditional” methods. The number of people using contraceptives increased from 40% in 1990 to 56% in 2002 and 61% in 2012. Usage dropped in 2017 to 51.8%, but this was almost entirely a result in the use of the male condom and ‘traditional’ methods. ^1 , 2^

Culture and religion appear to be significant determinants of family size in Jordan, with a preference for male children and religious beliefs that large families are required; this has a great impact on family planning decisions. ^3^ Moreover, familial pressure from husbands and in-laws strongly discourages the use of MFP methods. ^4^ More traditional family planning methods, while popular, are often applied with insufficient knowledge of how best to use the method. ^5^

Strategies directed toward lowering fertility and birth rates that are inexpensive and easy to administer are desired by governmental and international agencies. High quality family planning services that cover women’s needs are available in the governmental clinics in Jordan. ^2^

Although previous studies have reported findings from Jordan, they were quantitative in nature, which help to study the link between variables. ^3 - 5^ Quantitative design was not considered appropriate for this study because it fails to explain why such link exists. Furthermore, it may not help to obtain an understanding behind the women’s inappropriate use of MFP methods which qualitative designs offer. ^6 , 7^ Given the need to seek an understanding of the women’s perspectives regarding the use of MFP methods and services, qualitative design was considered appropriate. Understanding the specific needs, and the cultural and social factors that may influence women’s choices when using MFP methods and services can better inform family planning programmes. ^8^ In addition, qualitative design can be a powerful way to inform health care providers about women’s perspectives and needs, which can be used to promote practice change. ^9^ The purpose of this qualitative study, therefore, was to better understand women’s perceived barriers that might affect their ability to use family planning methods offered by the Jordanian Ministry of Health (MOH). 

## METHODS

A qualitative descriptive study design, using focus group discussions (FGD), was conducted to better understand the women’s perceived barriers to using MFP methods. The study was conducted in two states in the north of Jordan, namely Irbid and Mafraq states, which have a high population density. A list of the comprehensive health centers (HC) in selected districts was obtained from the Director of Primary Health Care at MOH. The comprehensive HCs in the districts of the two selected states were the primary sampling units. We purposefully selected 12 centers, which ended up with 7 because of the saturation. HCs in which rural and urban environments were covered for adequate representation of women were selected. Each HC has one maternal and child health clinic (MCH). The plan was to conduct one FGD in each MCH; however, saturation occurred after the seventh FGD (3 suburban areas, 4 rural areas). Saturation was reached when no new information was obtained in the interviews and data collection become redundant. ^10^ FGD sessions were conducted from January to February 2018. The number of participants in the FGDs ranged from 6-12, and the total number of all participating women was 52.

After obtaining ethical approval from the Institutional Research Board of Jordan University of Science and Technology and the MOH, a purposive sample of Muslim married women of reproductive age, who had used or were still using FP methods, was selected by the research team with the help of one of the MCH clinics’ midwives. Unmarried women or those who had never used any family planning methods were excluded. Our sample happened to be Muslims because they constitute 97.2% of Jordan population. ^11^ Group discussion sessions were held in a quiet surrounding at a convenient place for all participants inside the clinic and were audio- recorded with permission using a digital voice recorder. At the beginning of each FGD, one of the research team members worked as a group moderator and introduced the research team to the participants. The purpose of the study was then explained, and the participants introduced themselves. 

Data were collected using semi-structured interviews. An interview guide was developed by the research team to guide the FGD sessions. The interviews were conducted in the local Arabic language and moderated by an investigator with a PhD nursing degree whose role was to help to clarify some points as needed and create an environment that encouraged the participants to contribute to the group discussions. A second researcher from the research team was responsible for taking field notes such as general environment or participants’ attitudes. Each group discussion started with a general question about the participants’ experiences and perception of family planning, and the care they received, such as “What are the different methods you used or are still using to prevent pregnancy?”, and then gradually progressed to more specific questions, which emphasized the type of information we needed to collect from the research participants such as “Who makes decisions about your use of family planning method?”. The FGDs lasted about 60-80 minutes in duration. The participants did not receive incentives; however, snacks and refreshments were offered at the end of each FGD. 

All interviews were transcribed verbatim for analysis. Data were analyzed manually using thematic analysis according to the process described by Braun and Clarke through a coding process for identifying, analysing, and generating categories and themes. ^12^ The whole analysis process was done in its original Arabic language to maintain trustworthiness of the results, which could be lost by early and inaccurate translation. Translation into English was undertaken after theme generation by two members of the research team who were fluent in Arabic and English. Preliminary analysis was conducted after each interview to get a general impression of the data, which allowed for early identification of gaps in the interviews guide that sometimes required further clarifications from FGDs participants. 

Trustworthiness of the study was maintained through using four criteria: credibility, transferability, dependability, and confirmability. ^13^ To ensure credibility, we shared the results of the study with some participants (member checking) in order to confirm that the findings were reflective of their experiences. In addition, peer debriefing was maintained through the process of checking preliminary findings and interpretations against our raw data. Transferability refers to the degree to which the results of the study can be transferred to another setting or context. The researchers’ responsibility was to accurately describe the culture of the study; thus, the reader can judge how transferable the interpretations are to other situations and other cultural contexts. Dependability is concerned with the consistency and stability of the study over time and the possibility for another researcher to replicate the study. As to data consistency, all decisions that the researchers made during data collection and analysis were assessed and discussed through regular meetings with experienced qualitative researchers. Finally, to ensure confirmability, we maintained a detailed description of the research steps undertaken from the start for checking and reanalysis by others if needed. 

Ethical approval was obtained from the Institutional Research Board of Jordan University of Science and Technology (ethical approval reference number 26/94/2017) and the MOH. Consent forms were obtained from all participants, and at any point in the interview, if participation caused any discomfort for them, women were told that they had the right to withdraw from the study. While some women left the groups to attend medical appointment, none appeared to leave because of discomfort.

## RESULTS

A total of 7 FGDs were conducted. Of these FGDs, 3 were in suburban areas and 4 in rural areas. All participants were Muslims, with an age range of 22 to 50 years old, various levels of education ranging from primary school to a university degree (only one illiterate woman), and various number of children ranging from 1–9 (Table 1). Data analysis revealed three main themes and 6 subthemes (Table 2).

**Table 1:** Socio-demographic data of the participants (N=52)

Mother code	Age/ year	Number of children	Education level
P1	40	5	Bachelor
P 2	30	3	Secondary school
P 3	31	4	Bachelor
P 4	30	6	Secondary school
P 5	32	4	Bachelor
P 6	32	5	Bachelor
P 7	28	5	Bachelor
P 8	27	1	Secondary school
P 9	31	4	Bachelor
P10	27	2	Diploma
P11	40	4	Secondary school
P12	35	2	Diploma
P13	30	4	Secondary school
P14	27	2	Bachelor
P15	39	2	Diploma
P16	37	3	Diploma
P17	37	2	Bachelor
P18	45	7	Bachelor
P19	22	2	Secondary school
P20	23	2	Bachelor
P21	32	2	Bachelor
P22	40	6	Diploma
P23	37	5	Secondary school
P24	26	2	Secondary school
P25	50	5	Illiterate
P26	48	5	Diploma
P27	22	2	Secondary school
P28	28	1	Bachelor
P29	46	9	Primary school
P30	42	7	Primary school
P31	45	4	Diploma
P32	47	2	Bachelor
P33	40	8	Primary school
P34	34	6	
P35	23	4	Secondary school
P36	43	3	Bachelor
P37	32	4	Bachelor
P38	30	2	Primary school
P39	36	3	Diploma
P40	32	5	Bachelor
P41	25	1	Primary school
P42	27	2	Bachelor
P43	27	2	Bachelor
P44	28	3	Diploma
P45	31	4	Primary school
P46	32	4	Diploma
P47	41	3	Bachelor
P48	36	4	Primary school
P49	33	5	Secondary school
P50	26	5	Secondary school
P51	33	5	Bachelor
P52	28	2	Bachelor

**Table 2:** Extracted subthemes and themes from data

Subthemes	Themes
To conform to family and social pressure to bear children	Conforming to social and cultural norms
To prioritize having male children	
Need for consistency across providers in family planning counseling	Unmet needs in expected family planning counselling
Need for follow-up counseling	
Expected side effects	Undesirable side effects
Anticipated side effects	

### Theme 1: Conforming to Social and Cultural Norms

This theme describes the impact of social norms on the women’s decision of having children and, therefore, on their use of MFP methods. Subthemes that comprise this theme are the decisions ‘to conform to family and social pressure to bear children’, and ’to prioritize having male children’. 

### To Conform to Family and Social Pressure to Bear Children

Social norms and social networks influence perceptions about using MFP methods and the decision to have children. The majority of participants stated that the decision to use MFP methods was a shared one between wife and husband. However, they affirmed the family and social influences on their decisions on the use and on the method of MFP. Pressure to have children may come from the husband, the mother-in-law and/or the community in general. Differences existed between what women wanted and what their husbands wanted in terms of the total number of children they intended to have. According to the majority of participants, it is mostly a shared decision, but if agreement was not reached, the final decision is that of the husband. Participants affirmed that husbands and mothers-in-law were influential in decisions related to family size. A rural woman, who only wanted two children said, *“I wanted to have two, but because of the pressure from my husband and mother-in-law, I ended up with five.” (P26)*

There was no agreement among the participants about the one who makes the final decision on the total number of children. A few said that mothers-in-law were influential, but if the husband and wife had a mutually different opinion, they could resist the mother-in-law’s decision. One participant said, *“despite my mother-in-law’s pressure, my husband and I had the final decision on the size of our family”* (P37)

Discrepancies in the decisions to use MFP methods were noted between women from the different geographical locations of the research settings. A rural 46-year-old woman, for example, with 9 children, married at 17, began to use birth control after her 9th birth (twins) but felt guilty for using it because, according to her, *“If I did not use them, I would have more children to be proud of now.” (P29)*

A 42 mother of 7 children from the same FGD opposed the use of birth control methods and thought that *“women should have as many children as possible.” (P30)* In the suburban areas, birth spacing was totally acceptable by most women, which according to them *“allows you to be a better parent… so you can enjoy children and pay adequate attention to them.” (P23)*

The choice of women to use MFP methods is affected by many social and environmental factors that may reduce their ability to decide independently. As illustrated above, decisions regarding family size may affect the women’s choice of using MFP methods. Women also talked about the factors that affected their decision about the number of children they should have and, therefore, about their use of MFP methods. Some of these factors, such as the women’s relationship with their husbands, made them choose to have fewer children as they became discouraged when their marriage was threatened. According to one of them, *“children are the only reason to stay with a bad-mannered husband.” (P17)* On the other hand, having more children to discourage their husbands from taking another wife was another justification for having more children that women identified: *“If you don’t have enough children, he will marry another wife who starts having children for him” (P23)*

### To Prioritize Having Male Children

Not only the number of previous children affected the women choices to have more children, but also the gender of previous children was considered. Women recognized the benefits of family planning but often desired to have children soon after marriage and keep having children until male children were born for cultural reasons. Women stated social and cultural pressures to have a male child. According to the majority of them, if their children are all girls, birthing more children, hopefully boys, is recommended. If a woman only has one boy, she is also encouraged to have a second boy. One participant, for example, had problems giving birth: all her babies were premature, and she had three girls and one boy; she explained that *“My husband did not care that I suffer during my deliveries; he wanted me to keep trying to have another boy.” (P31)*

A 23-year-old participant stated because her first child was female, she was under pressure from her in-laws to get pregnant again shortly after she gave birth *“I wasn’t planning to have another baby soon, but they (in-laws) put so pressure on me to have a brother for my daughter” (P20)*.

## Theme 2: Unmet Needs in Expected Family Planning Counselling

The second theme explains some of the needs related to accessing appropriate family planning methods. Under this theme, women talked about the ‘need for consistency across providers in family planning counselling, and the ‘need for follow-up counselling’.

### Need for Consistency Across Providers in Family Planning Counselling

When women were asked about their satisfaction about the counselling process that they are receiving in the health centres (HC), they raised concerns about the inconsistent information and counselling given by various providers. Many women reported that in various HCs *“they would not give pills or insert IUDs until after the first child,” (P42)* and if the first child was a daughter, they were advised to use condoms. According to the women, there are no fixed guidelines on how to receive counselling, but rather it depends on the midwife’s attitude or willingness to do the counselling. Some women referred to the health centre with preferences and got what they asked for without counselling; others received full counselling. In another center, one woman claimed that the IUD was not given after her first baby at this clinic, but others said they got it at the same HC after their first pregnancy. 

Women expressed why they were satisfied with the counselling provided by the healthcare providers because of the *“availability of MFP methods,”(P49)* which are offered for free, *‘midwives are cooperative,’(P10)* and the HCs are accessible. On the other hand, they had a longer list of why they were not satisfied. Limited services were listed by the majority of them as some centres provided counselling on certain days of the week only, which is not convenient for their schedules. 

The length of counselling process varied in different centers and among midwives, and was not perceived as adequate for many women, as they believed that they needed more time and that *“midwives give the counselling hastily most of the time.” (P15)*. 

### Need for Follow-up Counselling

Participants stressed the need for follow-up counselling after receiving FP, which is currently lacking in the majority of HCs. They welcomed the idea of having a follow up communication and stated that the most preferred means of communication were social media group on Facebook or WhatsApp, for example, or through electronic messages. 

*“I have a very bad memory; I always wanted someone to remind me to take my pills. Sometimes I found myself with no pills at all. It would be great if they send me a massage a head of time to remind me of my appointment.” (P44)*

## Theme 3: Undesirable Side Effects

Another barrier to using MFP methods was the undesirable side effects. Subthemes that comprised this theme were ‘experienced side effects’ and ‘anticipated side effects’.

### Experienced Side Effects

All participants in this study were using or had used one or more of MFP methods. However, the majority of them reported experiencing one or many side effects themselves. The most common reported side effects of using IUDs included bleeding, headaches, ulcers, and ovarian cysts. 

“I used the IUD for two years, but I removed it because I experienced frequent bleeding that led to anemia. Now, I am using condoms and I am OK with them” (P22)

Pills caused nervousness, especially for those over the age of 35. Pills/shots/implants caused migraine, moodiness, irritability, obesity, and headache, and according to them, should be not used after the age of 35 or 40 years. One participant said *“I used the pills for only one year and couldn’t tolerate the side effects. I had severe headache and mood swings” (P27)*; another one added *“I am using the pills for the last 3 years and they are good, but I gained weight because of them”. (P34)*

A number of women said that these side effects had caused them to use more natural methods of birth control such as withdrawal: *“I have been experiencing too many side effects caused by different family planning methods, which made me think of using natural methods from now on.” (P40)*

Condoms are considered the safest method for women; however, they complained of their husbands’ dissatisfaction with them. 

### Anticipated Side Effects

According to the participants, not only did they experience side effects themselves, but acquaintances (friends, relatives) had similar side effects of using these MFP methods. In addition, they heard rumours of other potential side effects including infertility, cardiovascular problems such as heart attack, and early death.

“I did not want to use the IUD or the pills because I heard a lot about their negative health effects, and no one encouraged me to use them. Instead, I am using natural method more.” (P7)

Women were dissatisfied with the available family planning methods in terms of possible side effects. Their preference from highest to lowest were IUD, condom, pills, shots/implant, and finally natural methods (especially withdrawal). However, they believed that natural methods are not effective on their own. Most of them used withdrawal when they did not intend to use MFP because of the expected side effects, *“Natural methods are not guaranteed, but I have to use them because of many side effects caused by modern methods.” (P34)*

## DISCUSSION

This qualitative study aimed to help better understand the barriers, social norms, and behaviours that might affect the women’s use of family planning services in Jordan. Women were interviewed using FGDs approach to build an understanding of the current situation of contraceptive counselling process. As described in our results, using family planning in Jordan is affected by many underlying social and cultural factors that necessitate an in-depth understanding. Our findings indicate that social and cultural expectations educate women to strongly desire to have children and become mothers. This finding is congruent with other studies that reported that societal pressure and expectations played a role in the decision of having children within the first two years of marriage. ^14 , 15^ Jordanians highly value children and regard parenthood as a social must. This desire is rooted in the high rank human relations that a mother has in Islam. ^16^ It is also culturally and religiously important for Muslims to have many well-behaved children. ^17^ In fact, children are one of the main motivations for marriage in Islamic tradition. ^15^

The women in this study indicated how much social pressure influenced their decision to use MFP methods and have more children. Social pressure was also identified by the United States Agency for International Development (USAID) report as among the many reasons of Jordanian women for having more children. ^14^ Our results have suggested the importance of husbands and other family members in their decision-making processes. Men’s involvement in MFP contributes to accepting not only a contraceptive, but also its effectiveness and continuation. ^18 , 19^ This is consistent with the results from other cultures. For example, a previous study described how in Mexico, husbands take the final decision on using contraceptives with some influence from their mothers. ^20^ Chiapas possessed the lowest rate of contraception use among women 15−49 years old (44.6% For this reason, husbands’ involvement is very important to be considered when developing any family planning program. A key study in Jordan that evaluated men’s involvement in family planning counselling, in which 1300 women were randomized to receive woman only counselling, couple-counselling, or no counselling, found that couple counselling increased the use of contraceptives by 54% and improved the spousal communication. ^21^

Women also feel the need to have more children because of the worry of losing their husbands, which the participants in this study had emphasized. This may occur by divorce, or through marrying a second wife, as, according to religious and cultural beliefs, a husband of a childless woman has a good reason for practicing polygamy. ^22 , 23^ In addition to increasing the strength of the community, having many children increases the personal status of the family. Studies indicate that women in Arab countries are expected to become pregnant within the first month after marriage. Those who experience delayed pregnancy suffer many social and emotional consequences, including depression and disappointment. ^15 , 24 , 25^

Gender preference for boys is another major factor that contributes to the women’s use of MFP methods. As indicated in our findings, women desist using MFP when only having girls. Male preference is still a social and cultural norm in the Arab world generally and in Jordan particularly, which can partly be explained by the family’s desire for a boy who can alone keep the name of the family, increase of power and influence through having many boys, and importance of having males to secure the family financially. ^26 - 28^

The women in this study emphasized the need for consistency across providers in family planning counselling and follow-up counselling. In addition, concerns were reported about the side effects of MFP methods. While the lack of uniformity on receiving counselling and lack of follow-up contact were unique findings to our participants, the concern about side effects was reported by participants in studies in different countries, ^29 - 31^ which necessitate the need for addressing these concerns by the health care professionals and stressing the need to be explicit about the possible side effects when educating women, so they can make informed decisions. ^30 , 32^ Midwives should respect the woman’s choices and recognize that every woman is different. While some women do not experience side effects, they may be debilitating for others. Complaints should be taken seriously and alternatives should be sought if there is no other solution for them. The use of natural birth control methods, such as using fertility awareness methods with counting, temperature and the mucous method could be beneficial for many women. These methods, when combined with or replacing withdrawal and condoms that many women are using, could be very effective. ^33^

As with other studies, this study had several limitations. First, none of the HCs was located in villages with a primarily Christian population. Different perspectives may be gained from Christian participants, which may contribute to broader understanding of the phenomena. Second, the sample is limited to a specific geographical area; thus, the results are not generalizable. And finally, involving male participants may produce better understanding.

## CONCLUSION

This study identifies a number of barriers that Jordanian women perceived for not using MFP methods. These included conforming to the social pressure, inconsistency of the counselling process, and undesirable side effects. The data obtained from this study could be used to develop a training manual for the midwives designed to overcome barriers to using MFP methods, and to enhance the counselling process in HCs in Jordan. The delivery of information related to the use of MFP methods needs to be socially, religiously, and culturally acceptable for the users. Therefore, these factors should be carefully addressed in any developed counselling programs. Studies are needed about the influence of service providers on the couples’ preferences of certain contraceptive methods and their general uptake of MFP methods, as well as the effect of following up with text massage on the continuity of using MFP methods.
